# A Machine Learning Framework Predicts the Clinical Severity of Hemophilia B Caused by Point-Mutations

**DOI:** 10.3389/fbinf.2022.912112

**Published:** 2022-06-23

**Authors:** Tiago J. S. Lopes, Tatiane Nogueira, Ricardo Rios

**Affiliations:** ^1^ Center for Regenerative Medicine, National Center for Child Health and Development Research Institute, Tokyo, Japan; ^2^ Institute of Computing, Federal University of Bahia, Salvador, Brazil

**Keywords:** hemophilia B, protein structure, machine learning, bioinformatics, residue network, FIX, FIXa

## Abstract

Blood coagulation is a vital physiological mechanism to stop blood loss following an injury to a blood vessel. This process starts immediately upon damage to the endothelium lining a blood vessel, and results in the formation of a platelet plug that closes the site of injury. In this repair operation, an essential component is the coagulation factor IX (FIX), a serine protease encoded by the F9 gene and whose deficiency causes hemophilia B. If not treated by prophylaxis or gene therapy, patients with this condition are at risk of life-threatening bleeding episodes. In this sense, a deep understanding of the FIX protein and its activated form (FIXa) is essential to develop efficient therapeutics. In this study, we used well-studied structural analysis techniques to create a residue interaction network of the FIXa protein. Here, the nodes are the amino acids of FIXa, and two nodes are connected by an edge if the two residues are in close proximity in the FIXa 3D structure. This representation accurately captured fundamental properties of each amino acid of the FIXa structure, as we found by validating our findings against hundreds of clinical reports about the severity of HB. Finally, we established a machine learning framework named HemB-Class to predict the effect of mutations of all FIXa residues to all other amino acids and used it to disambiguate several conflicting medical reports. Together, these methods provide a comprehensive map of the FIXa protein architecture and establish a robust platform for the rational design of FIX therapeutics.

## 1 Introduction

In humans and other animals, the blood is responsible for functions essential to sustain life, including the transport of gases and nutrients, regulation of body temperature, and importantly, the repair of damaged blood vessels (i.e., clotting). This process involves the activation and adhesion of platelets and fibrin to form a platelet plug that ceases the blood loss (Lee et al., 2014; Hoffbrand et al., 2016). The blood coagulation pathway consists of a well-orchestrated series of protein activation and complex formation, and involves more than 10 different components, termed coagulation factors (Lee et al., 2014; Hoffbrand et al., 2016). Disruptions of this delicate system often leads to hemorrhage or thrombosis.

Among these blood coagulation disorders is hemophilia B (HB), a relatively rare condition affecting 1 in ∼50,000 live births, caused by mutations in the coagulation factor 9 gene (F9) (Lee et al., 2014). The human F9 gene is located on the X chromosome, has 34 kb and after transcription it produces an 8-exon mRNA molecule encompassing 2,802 bp. The encoded protein has 461 amino acids, and after removal of the 46 residues signal- and leader peptides, a 415 mature protein is produced (Anson et al., 1984). Following an injury and the consequent trigger of the coagulation cascade, FIX is activated on a two-step calcium-dependent operation by the FVIIa/Tissue Factor complex and by FXIa. These activation steps result in the removal of a peptide spanning residues 192-226, and produce a FIXa with a light- and a heavy-chain [residues 47-191 and 227-461, respectively (Di Scipio et al., 1978; Bajaj et al., 1983)].

The FIXa protein has four domains (Gla, EGF1, EGF2, and serine protease). The Gla domain is involved in binding the phospholipid membrane of platelets (Rawala-Sheikh et al., 1992), the tandem copies of the EGF domain are involved in binding other coagulation factors (Wilkinson et al., 2002; Zhong et al., 2002), and the serine protease domain (SP), which comprises about half of the FIXa’s mass, contains the amino acid triad responsible for the FX activation (Brandstetter et al., 1995).

Although FIX exhibits a relatively simple domain architecture, substitutions of amino acids often lead to the disruption of the FIX catalytic activity, as indicated by more than 1,000 mutations reported to date (Rallapalli et al., 2013). These mutations cause HB with different symptoms (White et al., 2001), ranging from mild cases with only occasional bleeding episodes (5%–40% of the normal FIX activity), to moderate (1%–5% of the normal FIX activity), and severe cases (less than 1% of the normal FIX activity).

For patients who have access to treatment, it consists of periodic prophylactic injections of recombinant FIX to prevent bleeding episodes. Recently, clinical trials have demonstrated the feasibility of gene therapy, whereby an adenovirus was modified to introduce the F9 gene in the liver of patients to generate a steady production of FIX (George et al., 2017). Although these treatment options dramatically improved the quality-of-life of HB patients, the development of inhibitory antibodies in 1.5%–3% of patients (Santoro et al., 2018), the short half-life of recombinant products (Franchini et al., 2013), and the toxicity associated with the high doses necessary for efficient gene therapy (Manno et al., 2006), indicate that HB therapeutics still require further improvements.

In this sense, as attested by the fact that some FIX mutants—either natural (Simioni et al., 2009) or engineered (Nair et al., 2021)—dramatically increase the catalytic activity and the half-life of FIX, it is clear that a deep understanding of the FIX structure and function is crucial to accelerate the development of more potent and less immunogenic FIX constructs.

To address this issue, we created an in silico network representation of the FIXa structure—a residue interaction network (RIN)—where each of its residues is a node, and two nodes are connected by an edge if they are in close proximity to each other in the FIXa 3D structure. As we reported previously for FVIII (Lopes et al., 2021a; Lopes et al., 2021b), this novel representation allowed us to calculate several centrality measures of each amino acid, effectively quantifying their importance in the FIXa structure and indicating which amino acids are more or less tolerant to substitutions. To ensure the robustness of this approach, we carefully validated our in silico findings against hundreds of clinical reports associating mutations to the severity of the HB symptoms.

Next, we created an open-source machine learning framework called HemB-Class to generalize these findings and predict the effects of mutations of all FIXa residues to all 19 remaining amino acids. Notably, we verified that we could use the HemB-Class framework to disambiguate clinical reports that had conflicting results (i.e., database entries showing different severities associated to the same amino acid substitution).

Together, the findings presented here provide a comprehensive map of the FIXa protein structure, and demonstrate the feasibility of in silico tools to mechanistically quantify the characteristics of every residue of this vital coagulation factor.

## 2 Results

### 2.1 Creation of the FIXa RIN

The FIXa protein activates FX to FXa via a protealytic cleavage mediated by its amino acid triad located on the SP domain (Anson et al., 1984; Johnson et al., 2010), and this reaction is catalyzed several thousand folds by the presence of FVIIIa (Lee et al., 2014). To understand the details of this process and to design efficient recombinant therapeutic proteins, it is essential to investigate the protein structure of FIXa. Even though no complete structure of the human FIX was determined, the structures of its individual domains (Freedman et al., 1995; Rao et al., 1995; Johnson et al., 2010) and the complete porcine (Brandstetter et al., 1995) version of this protein were determined at good resolutions (1.7Å–2.8 Å). Therefore, to study the structure of FIXa we used the same strategy from a previous study that aligned the individual domains of the human FIX to the backbone of the porcine structure, and further refined it using optimization software (Rallapalli et al., 2013). We verified that in this structure, ∼30% of its residues are buried at the core of the structure, 15% present in alpha helices, 24% in beta-sheets, and more than 40% are present in loops of different shapes and sizes (Supplementary Table S1). Compared to the human FIX structure predicted by AlphaFold 2 (Tunyasuvunakool et al., 2021), the two models displayed an almost identical conformation on the heavy chain, but had considerable differences on their light-chains; this is due to the fact that the AlphaFold model was based on the complete form of the FIX protein and had low modeling confidence at several regions (Supplementary Figure S1). Given that the model we use in this study is derived from structures determined at good resolutions (1.7Å–2.8 Å), we opted to use solely our model for the analyses. Although this homology model was a “snapshot” of this coagulation factor and did not take into account the conformational changes that take place upon interaction with its partners (Freato et al., 2020), this domain alignment strategy yielded an appropriate homology model of FIXa, with more than 95% of its residues displaying good or very good conformations (Supplementary Figure S2), and was successfully used in protein binding studies (Venkateswarlu, 2010; Venkateswarlu, 2014).

**FIGURE 1:**
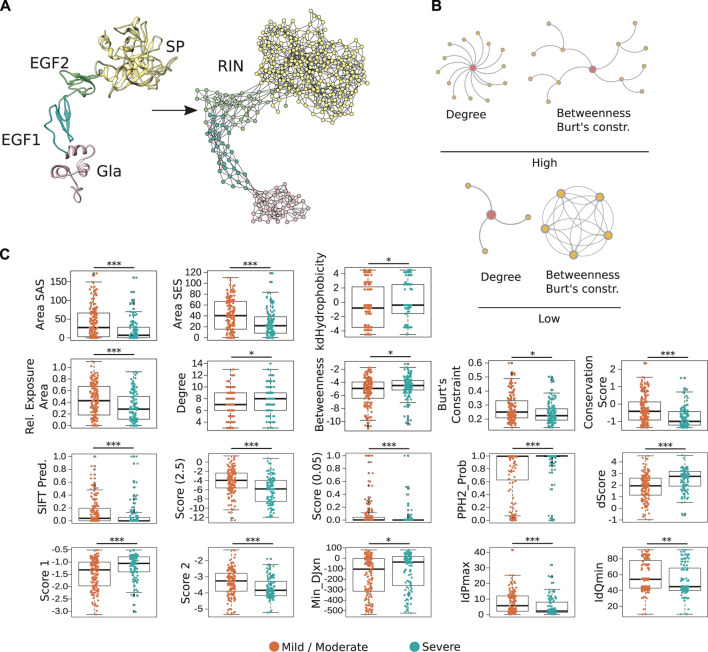
Representation of FIXa structure as a residue network. **(A)** In the FIXa RIN, each node represents an amino acid, and two nodes are connected by an edge if their atoms are in close proximity (∼5 Å). **(B)** The degree quantifies the number of connections a residue has, the betweenness indicates how many times a node served as a bridge on the shortest path along two other amino acids, and the Burt’s constraint was derived from social science studies to quantify the position of advantage of individuals within an organization (Burt, 2009). Nodes with high-degree participate in multiple molecular interactions, and those with high-betweenness and low Burt’s constraint serve as intermediate between different groups of amino acids. In contrast, residues with low degree, low betweenness and high Burt’s constraint usually do not have many connections to other residues and are located at the periphery of the network. **(C)** Properties derived from the FIXa structure or from the RIN are good indicators of the severity of HB. Depicted is the solvent-accessible (areaSAS) and the solvent-excluded (areaSES) surface areas, the relative exposure of amino acids (Rel. Exposure Area), the conservation of the FIXa residues (smaller values indicate higher conservation), and the RIN centrality measures. Also depicted are measures derived from SIFT (Sim et al., 2012), Provean (2 Scores, −2.5 and 0.05) (Choi and Chan, 2015), and from Polyphen-2 (PPH2-Prob, dScore, Score 1, Score 2, MinDJxn, IdPmax, IdQmin) (Adzhubei et al., 2010). The boxplots show the median (center line), the first and third quartiles (lower- and upper-bounds), and 1.5 times the inter-quartile range (lower- and upper whiskers). Each dot in the plot is an amino acid mutation (i.e., a clinical case report). Unpaired, two-sided Wilcoxon test (***p-values < 0.001; **p-value < 0.01; *p-value < 0.05).

Next, given that protein structures are chains of amino acids organized in a three-dimensional space, we reasoned that a network representation of the FIXa structure could offer a detailed understanding of its underlying properties. Therefore, we created a RIN where each of its amino acids is a node, and two nodes are connected by an edge if they are in close proximity to each other in the FIXa 3D structure (Methods). The edges between nodes indicate that there is either a 1) side-chain–side-chain, 2) side-chain–main-chain, 3) main-chain–main-chain hydrogen bond or non-covalent interaction between the atoms of the residues (usually located within less than ∼5 Å) (Figure 1A; Supplementary Table S2 contains the complete network). Interestingly, the FIXa RIN displayed several hydrogen bonds between residues (Supplementary Figure S3); in all domains, these residues were separated by ∼6 Å, and these bonds most likely help stabilize the FIXa structure by maintaining the correct orientation of all interacting partners.

In total, the FIXa RIN had 360 nodes and 1,229 edges. Previous studies demonstrated that the centrality measures of amino acids in a RIN are strong indicators of the protein stability (Dokholyan et al., 2002; Yan et al., 2014; Nisthal et al., 2019) and provide valuable information about the role of residues in the protein conformation and interaction with other proteins (Reichmann et al., 2005; del Sol et al., 2006). Therefore, to quantify the centrality of the FIXa RIN, we calculated several measures based on distinct underlying principles (Figure 1B), as well as quantitative features derived directly from the protein structure, like the solvent exposed area, hydrophobicity and the psi and phi angles of each of its residues, and their conservation throughout evolution (Methods).

Next, we wondered if these features could be used as indicators of the severity of HB. Similar to other proteins (Kessel and Ben-Tal, 2010), we found that mutations of the most conserved, hydrophobic and buried residues are usually associated with severe forms of HB (Figure 1C). Moreover, we evaluated the predictive performance of 3 popular methods that are able to determine the effect of point mutations in proteins [i.e., Provean (Choi and Chan, 2015), Polyphen-2 (Adzhubei et al., 2010), SIFT (Sim et al., 2012)]. These methods output a binary classification of the most likely result of amino acid substitutions (i.e., neutral or deleterious), and numerical scores quantifying this effect. We used 393 FIXa mutations as input to these methods and verified that while their binary classifications could not predict the severity of hemophilia A (Supplementary Figure S4), their numerical scores were powerful discriminators of severe and mild/moderate cases (Figure 1C). Finally, we observed that substitutions of the most central residues of the FIXa RIN (indicated by the high-degree, high-betweenness and low Burt’s constraint values), lead to more severe symptoms. On the other hand, mutations of the less conserved residues located at the protein surface, and less central in the FIXa RIN are mainly related to mild or moderate HB symptoms (Figure 1C).

In an analogy to the train system of a large city, disturbances at the “hub” stations quickly propagate and cause the collapse of the transportation network; in a similar fashion, substitutions of the most connected residues of FIXa lead to the impairment of its function. As our results demonstrate, the FIXa RIN is able to capture the underlying properties that associate the position of each network residue to the severity of HB symptoms, and together with existing methods, these findings converge from multiple lines of evidence.

### 2.2 RIN Derived Measures Identify Critical RIN Residues

After verifying the suitability of the RIN to represent the FIXa structure and to quantify the importance of its residues, we aimed to identify which of them are critical for the proper functioning of this protein.

**FIGURE 2:**
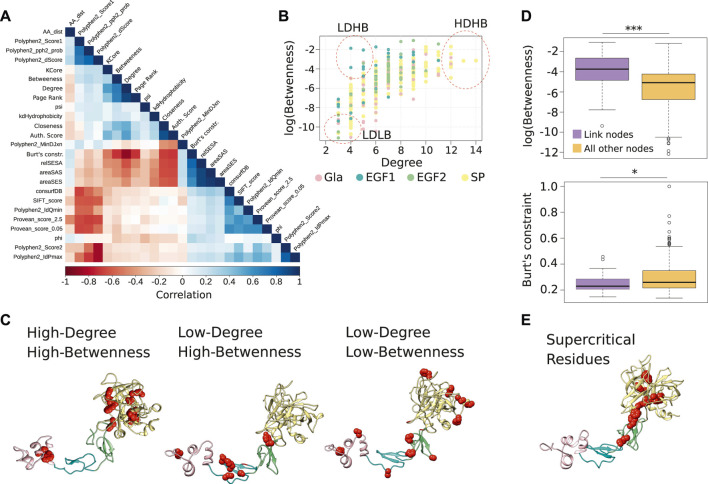
Centrality measures from the FIXa RIN and the important residues. **(A)** The Spearman correlation between all measures considered in this study. **(B)** The degree and betweenness of all residues of the FIXa RIN. Each dot represents an amino acid, and groups of residues with different characteristics are highlighted. **(C)** The location of the residues highlighted in panel **(B)** in the FIXa protein structure. **(D)** The boxplot displays the betweenness and the Burt’s constraint values of the nodes taking part in atomic interactions with residues in other domains of FIXa (link nodes), compared to the nodes interacting only with residues from the same domain. **(E)** The residues with the highest degree, betweenness and (lowest) Burt’s constraint; these residues are most likely the most central of the whole FIXa protein. The boxplots show the median (center line), the first and third quartiles (lower- and upper-bounds), and 1.5 times the inter-quartile range (lower- and upper whiskers). Unpaired, two-sided Wilcoxon test (***p-values < 0.001; *p-value < 0.01; *p-value < 0.05).

For this purpose, we compared the measures we calculated for the FIXa RIN, and found that several of them were correlated (Figure 2A). Therefore, for further analyses we selected only the least correlated measures: two well-studied centrality measures (degree and betweenness), and an index not commonly used in biological network analysis [the Burt’s constraint, derived from social science studies (Burt, 2009)].

After inspection of the degree and betweenness values of residues in the FIX RIN (Figure 2B), we divided the residues into three patterns, 1) High-Degree and High-Betweenness (HDHB), 2) Low-Degree and High-Betwenness (LDHB), and 3) Low-Degree and Low-Betwenness (LDLB) (Table 1). We found that the HDHB residues are mainly part of alpha helices or beta-strands, are connected to 12–14 other residues *via* non-covalent or hydrogen bonds, are buried at the core of the Gla and the SP domains, and although mutations at these residues are mainly associated to severe forms of HB, occasionally there are reports of moderate symptoms (Figure 2C). Among these residues, Phe424 is located at the edge between the SP and the EGF2 domains, and is consistently associated with severe forms of HB if mutated to leucine, valine, or serine (Chen et al., 1991; Caglayan et al., 1997; Liu et al., 2000). Moreover, we found that in the FIXa RIN, 40 residues take part in atomic interactions with residues from a different domain. Interestingly, while the degree of these residues did not differ from other residues of FIXa, their betweenness and their Burt’s constraint values were markedly different—they were more than 3 times higher compared to residues interacting only with residues from the same domain (Figure 2D; Supplementary Table S3).

**TABLE 1:** Key-residues identified using the centralities of the FIX RIN.

Group	Pos.^a^	AA	Dom.	Rel. Exp. Area^b^	Degree^b^	Betw.^b^	Burt’s Constr.^b^	Conserv.^c^	Severity^d^			Struct.^e^
									Sev.	Mod.	Mild	
**HDHB**	87	Phe	Gla	31.1	99.4	92.8	11.4	8	3	2	1	H
	88	Trp	Gla	35.3	99.4	86.1	16.4	8	1	—	—	H
	240	Trp	SP	25.3	99.4	95.6	3.9	8	7	4	1	T
	256	Ile	SP	6.4	100.0	91.1	2.5	8	1	—	—	E
	282	His	SP	11.1	99.4	85.6	3.3	5	1	—	3	T
	316	Ile	SP	7.5	99.4	82.8	0.3	8	7	4	—	C
	358	Arg	SP	41.7	99.4	80.6	9.2	3	1	—	1	T
	410	Asp	SP	30.6	99.7	90.0	1.1	9	7	4	—	T
	424	Phe	SP	9.7	99.4	99.2	2.2	2	7	—	—	E
	444	Tyr	SP	6.4	99.4	88.6	0.6	9	—	1	—	E
	450	Tyr	SP	10.5	99.4	83.6	6.7	6	4	1	1	H
**LDHB**	48	Asn	Gla	64.7	29.2	89.2	74.7	6	2	8	1	C
	97	Cys	EGF-1	51.1	29.2	81.9	78.6	9	5	1	2	T
	107	Ser	EGF-1	75.8	18.3	98.3	73.1	1	—	—	—	E
	109	Lys	EGF-1	57.8	29.2	96.9	75.8	3	—	—	—	E
	111	Asp	EGF-1	68.6	29.2	97.2	72.8	2	—	—	—	T
	125	Gly	EGF-1	59.4	18.3	79.4	80.6	8	4	—	—	T
	161	Tyr	EGF-2	67.5	29.2	87.5	35.3	7	2	2	—	T
**LDLB**	47	Tyr	Gla	100.0	0.8	3.3	74.7	5	—	—	—	C
	81	Thr	Gla	90.0	7.8	3.3	78.6	1	—	—	—	C
	99	Ser	EGF-1	96.9	7.8	3.3	73.1	5	—	—	—	T
	149	Ala	EGF-2	98.6	7.8	3.9	75.8	1	—	—	—	T
	160	Gly	EGF-2	99.7	0.3	3.3	72.8	8	5	10	4	T
	165	Glu	EGF-2	91.7	7.8	3.3	80.6	2	—	—	—	T
	171	Glu	EGF-2	75.0	7.8	4.4	35.3	8	—	—	—	C
	271	Thr	SP	94.4	7.8	3.3	98.9	1	—	—	—	T
	303	His	SP	81.7	7.8	3.3	98.3	5	1	—	1	T
	309	Ile	SP	93.3	7.8	3.3	99.2	1	—	—	—	T
	360	Phe	SP	90.8	7.8	3.3	96.1	2	1	1	1	T
	387	Lys	SP	83.6	7.8	3.3	96.9	2	4	—	5	T
	421	Gly	SP	97.2	0.8	3.3	99.7	3	—	—	—	T
**Super-critical**	144	Phe	EGF-2	8.3	88.9	100.0	10.8	4	—	—	—	E
	168	Lys	EGF-2	40.8	96.7	99.4	8.3	4	—	—	—	C
	256	Ile	SP	6.4	100.0	91.1	2.5	8	1	—	—	E
	263	Val	SP	14.7	96.7	96.7	1.7	6	1	—	—	E
	316	Ile	SP	7.5	99.4	82.8	0.3	8	7	4	—	C
	356	Trp	SP	33.1	96.7	91.7	1.4	9	9	3	4	C
	410	Asp	SP	30.6	99.7	90.0	1.1	9	7	4	—	T
	424	Phe	SP	9.7	99.4	99.2	2.2	2	7	—	—	E
	425	Leu	SP	14.4	93.6	98.1	0.8	7	1	3	1	E
	444	Tyr	SP	6.4	99.4	88.6	0.6	9	—	1	—	E

a Numbering following the Human Genome Variation Society numbering (HGVS).

b Percentile values, showing the percentage of other residues with centrality values smaller than the value indicated.

c Conservation according to the ConsurfDB server. It varies from 1 (least conserved) to 9 (most conserved).

d Number of reports in the EAHAD FIX mutation database, including all types of mutations. Visited in Feb. 2021.

e Secondary structure elements: alpha helix (H); beta-strand (E); coil (C); turn (T).

The LDHB residues are located at loops and beta-strands, but serve as bridges along the shortest paths between other amino acids. These residues are located mainly at the outer regions of EGF1 and EGF2 and have neighbors at different domains, most likely stabilizing the overall FIXa conformation. Most mutations at these residues are associated to severe forms of HB (e.g., Gly125Arg (Caglayan et al., 1997); however, residues Ser107, Lys109, and Asp111 had no reports of HB, possibly because these mutations occur in humans but people carrying this mutation did not show symptoms, given that these amino acid positions are not conserved and accepted different types of amino acids throughout evolution (Supplementary Table S1).

Next, the LDLB residues are the most peripheral residues of FIXa, located at the most outer loops of the Gla, EGF1, EGF2, and SP domains (Figure 2C). While six of those residues did not have HB reported in the medical literature, the remaining had a few cases described, indicating mainly mild, and occasionally moderate or severe cases (e.g., Thr271).

Along these lines, we also verified that the Padua (Simioni et al., 2009) and the CB 2679d-GT mutants (Nair et al., 2021), known to considerably increase the activity of FIXa, have a high surface exposure and centrality values tending towards the least connected residues of the FIXa RIN. Interestingly, we observed that while most FIXa surface residues interacting with FVIIIa display low connectivity and high surface exposure, some residues eluded this trend by taking part in multiple molecular interactions with other residues (i.e., Lys347, Arg379, Leu383), and caused a major reduction of FXa generation if mutated to alanine (Kolkman et al., 1999; Bajaj et al., 2001). This suggests that although most surface residues have low centrality values in the FIXa network, some are highly connected, most likely to preserve their correct positioning within the binding sites (Reichmann et al., 2005; del Sol et al., 2006).

Together, these findings indicate that centralities measures derived from the RIN help to identify critical residues of the FIX structure, and their position within the network largely reflect the severity of HB symptoms that ensues if those residues are mutated.

Having observed that the Burt’s constraint could significantly differentiate between HB severities (Figure 1C), and that this measure had only a moderate correlation with the betweenness centrality (0.78, Spearman correlation, *p*-value < 0.01), we considered it together with the degree and the betweenness and used the Pareto front to identify the FIXa residues that had the highest values on all three measures. This strategy offered a strong combination of local and global centrality measures, pointing to the residues that played a critical role in its local neighborhood (high degree) as well as on distal locations of FIXa (high betweenness and low constraint values)—we termed these residues supercritical (Figure 2E; Table 1).

We found that these residues are mainly part of beta-strands, are located in the hydrophobic core of the SP domain, at a surface loop of EGF2, and within less than ∼1.5 Å of several residues of EGF1. Moreover, the substitution of these residues is strongly associated to severe HB symptoms—for instance, Trp356, Asp410, and Phe424 had combined more than 30 reports of severe HB in the EAHAD database (Rallapalli et al., 2013).

In all, these findings indicate that the connectivity of residues in a RIN provides quantifiable information reflecting their importance, and the combination of measures can uncover patterns that would otherwise remain obscured.

### 2.3 Development of a Machine Learning Classifier for Hemophilia B

After identifying the properties of key FIXa residues, we wondered if we could use all structural and centrality measures in conjunction to train a machine learning classifier to predict the severity of HB that ensues upon mutation of the FIXa residues. A machine learning classifier algorithm works by learning the patterns from only a part of the input dataset, and by repeatedly tuning its parameters to prioritize features that are informative to predict the class of each instance. In our case, we had 393 instances representing a FIXa mutation that caused HB in a patient (213 mild/moderate, 180 severe). Current databases do not have enough data of Type I and Type II mutations to allow us to create specific machine learning models for each type (namely, those that impair secretion and activity, or those affecting only the activity of FIXa).

**FIGURE 3:**
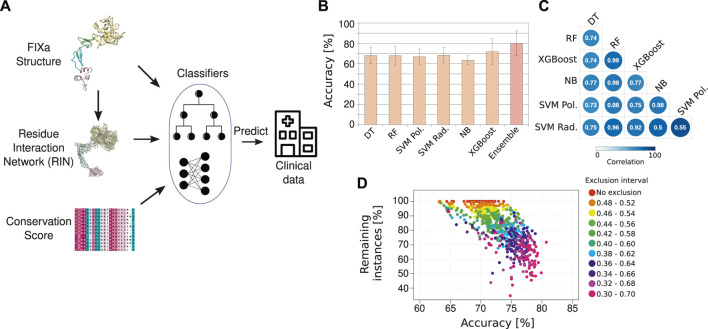
The HemB-Class machine learning framework. **(A)** Our machine learning classifiers received as input the properties from the FIXa structure, from the FIXa RIN, the conservation score of each amino acid and measures derived from other variant prediction algorithms [SIFT (Sim et al., 2012), Provean (Choi and Chan, 2015), and Polyphen-2 (Adzhubei et al., 2010)]. The output of our classifiers is the severity of HB, derived from clinical reports from the EAHAD FIX mutation database (Rallapalli et al., 2013). **(B)** Comparative performance of six classifiers and a combination of the best classifiers (Ensemble—we named it HemB-Class). The bars depict the mean values of 10 repetitions of 10-fold cross validations and the error bars are the standard deviation values. **(C)** Spearman correlation of the predicted probabilities outputted by the classifiers. **(D)** The trade-off between the number of instances classified and the accuracy. Each dot is the classification performance of an individual classifiers or the ensembles when we vary the classification threshold to create an “exclusion area” to disregard instances with ambiguous classifications.

The input features were the structural and centrality measures derived from the FIXa RIN and from the structure itself, the residue conservation score derived from a large multiple sequence alignment, and other measures of difference between the wild-type and the new amino acid after the substitution (Methods). Finally, the class label to be predicted was the severity of the disease (mild/moderate or severe) (Figure 3A).

We compared the predictive power of 6 different classifiers. The input data was divided into 10 equal parts, and 9 parts were used for training and 1 part was used to evaluate (test) the performance of the classification—namely, how many instances were correctly classified as mild/moderate or severe HB. During the training phase, the hyperparameters used to control the learning process were tuned using a 10-fold cross-validation approach to avoid overfitting the models. To increase the robustness of the results, this procedure was repeated 10 times to ensure that several combinations of training and test sets were considered. Using this approach, the classifiers obtained an overall accuracy of ∼70%, indicating that the individual algorithms could learn moderately well the underlying patterns of the FIXa structure and correlate them to the severity of HB symptoms (Figure 3B).

We observed that the classifiers outputted slightly different predictions for the same instance, as reflected in the low correlation between their outputted probabilities (Figure 3C). This situation is ideal for the creation of an ensemble of classifiers–i.e., the combination of predictions from different classifiers to come closer to the real class of an instance (Dong et al., 2020). Therefore, to find the best combination of classifiers, we calculated the median of their outputted predictions considering all possible classifier combinations, from individual algorithms to all six algorithms together. Additionally, we verified that the classification accuracy improved considerably if we created an “exclusion zone” where we did not consider instances that had ambiguous classifications (Figure 3D).

The best ensemble was the combination of two well-known algorithms, namely, Random Forest (Breiman, 2001) and XGBoost (Chen and Guestrin, 2016). We named this ensemble HemB-Class. Additionally, instead of outputting a simple binary classification (e.g., mild/moderate or severe HB), we implemented HemB-Class to output a probability that a mutation will impair the function of the FIXa protein. We named this output as Severity Score and verified that it raised the accuracy of HemB-Class to more than 80% while retaining more than 70% of the instances (Figure 3D). Additionally, HemB-Class achieved sensitivity of 0.71 and specificity of 0.89, demonstrating that it could accurately distinguish between mild/moderate and severe HB cases (Supplementary Table S4).

Together, these results indicate that although HB had only a few hundred unique instances—as is often the case for rare diseases—we found that a rational combination of classifier algorithms leveraged HemB-Class’ performance and led to correct predictions of the association between amino acid substitutions and the severity of HB. Importantly, the Severity Score provided the flexibility to select the stringency of the classification, either classifying more instances with less confidence, or less instances with more certainty.

### 2.4 Predicting the Severity of all Possible FIX Mutations

One of the greatest assets of machine learning classifiers is their ability to predict the class of instances not used during the training phase. For this reason, we aimed to predict the severity of 1,373 HB mutations that had conflicting clinical reports (i.e., mutations at the same position, but with different symptoms).

**FIGURE 4:**
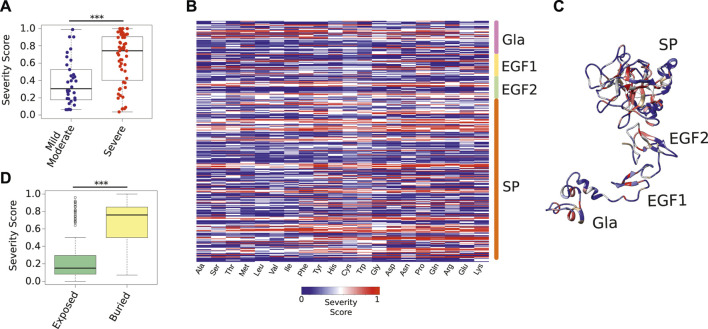
Severity Score of all possible FIXa mutations. **(A)** The Severity Score of mutations not used during the training phase because they had conflicting symptoms reported in the medical literature. Our predictions agreed with the majority class of each mutation (Supplementary Table S5). **(B)** The Severity Score predicted by the HemB-Class framework for the mutations of each FIXa residue to the 19 remaining amino acids. **(C)** The location of the residues with the highest Severity Scores. These residues, located at the core of each FIXa domain, are unlikely to accept any amino substitution (Supplementary Table S6). **(D)** The most buried residues (less than 25% relative surface exposure), have significantly higher Severity Scores than the most exposed residues. The boxplots show the median (center line), the first and third quartiles (lower- and upper-bounds), and 1.5 times the inter-quartile range (lower- and upper whiskers). Each dot in the plot is an amino acid mutation (i.e., a clinical case report). Unpaired, two-sided Wilcoxon test (***p-values < 0.001; **p-value < 0.01; *p-value < 0.05).

We used HemB-Class to calculate the Severity Score of those mutations and found that our predictions largely agreed with the majority class of the reports—i.e., mutations with high Severity Scores are often associated to severe forms of HB and vice-versa (Figure 4A). These results indicate that HemB-Class can help disambiguate FIXa mutations, and narrow down the number of candidates that require a meticulous laboratory follow-up (Supplementary Table S5).

Next, we used HemB-Class to predict the effect of mutating all residues of FIXa to all 19 remaining amino acids (Figure 4B). We found that the core of all domains have regions that are intolerant to mutations. For instance, Glu72 and Glu73 on the Gla domain, Cys97 on EGF1, Cys141 on EGF2, and Trp261 on SP were predicted to impact the function of FIXa if mutated to most other residues, especially if the changes introduce residues with opposite charges or differing sizes (Supplementary Table S6).

Finally, we combined all predictions to identify the residues that were the most reactive to mutations—in other words, residues unlikely to accept any amino acid substitution (Figure 4C). We found that the buried residues of all domains are the regions where mutations are more likely to cause a disruptive effect; however, while previously this was only a qualitative measure in the protein structure field (i.e., buried or exposed), we effectively represented this characteristic in quantitative terms and observed a major difference between the predicted Severity Score of the most buried compared to the exposed residues (Figure 4D), suggesting that substitutions aimed at improving the activity of FIXa should take the buried and exposed thresholds into account.

In summary, these results demonstrate that a versatile machine learning framework can anticipate the effects of amino acid substitutions in FIXa, as demonstrated by the close agreement between these predictions and hundreds of clinical reports collected over the past 3 decades.

## 3 Discussion

In this study, we established a novel representation of the FIXa structure that enabled us to quantify the importance of all of its amino acids. We organized the FIXa structure as a residue network, where two nodes are connected if they are in close proximity to each other in the 3D structure of the FIXa protein. We found that the amino acid centrality measures derived from this network are good indicators of the severity of HB that ensues if those residues are mutated (Figure 1). Moreover, we inputted these and other structural measures into a machine learning classifier framework that we named HemB-Class, and found that it accurately predicts the severity of HB. We used this framework to disambiguate conflicting medical reports and to predict with high accuracy the extent of impairing mutations.

In comparison to FVIII, the FIX protein is relatively simple, containing 461 residues and only 4 domains. However, this simplicity also requires that all of its binding sites and catalytic regions are correctly positioned for its activity to take place (i.e., activate FX) (Lee et al., 2014; Hoffbrand et al., 2016). While major perturbations to the F9 gene are more likely to result in severe HB (e.g., deletions or introduction of premature stop codons), the effects of single-nucleotide polymorphisms are less predictable. Therefore, as the conformation of proteins is intimately related to their functions (Kessel and Ben-Tal, 2010), studying the impact of amino acid substitutions in the overall protein architecture is an essential step to link and anticipate the effects of nucleotide substitutions to the clinical symptoms of HB.

In addition to identifying properties and centrality measures associated to mild, moderate and severe HB (Figure 1C), we used the FIXa RIN to identify the residues that display relevant properties and that can either be safely substituted, or should be avoided in attempts to improve FIX’s activity and immunogenic profile (Supplementary Table S1). In particular, we observed that the positions mutated in the Padua (Simioni et al., 2009) (Arg384) and the CB 2679d-GT (Arg364, Arg384, Thr389) variants (Nair et al., 2021), known to increase FIX’s activity and stability, have a centrality profile similar to other peripheral nodes (Figures 2B–D), corroborating the idea that residues with this profile can be safely substituted without impacting the stability of FIXa. Notably, we verified a mixed composition of more and less connected residues at the FVIIIa-FIXa binding sites (Kolkman et al., 1999; Bajaj et al., 2001), suggesting that while some residues can be safely substituted, others take part in numerous molecular interactions that hold the modular structure of the binding sites in place (Dokholyan et al., 2002; Reichmann et al., 2005).

While individual structural properties and the RIN measures are good to identify critical FIXa residues, we wondered if we could leverage the overall predictive power of our approach if we used machine learning algorithms (Figure 3). Indeed, we found that after a strict training, evaluation and combination procedure, we could use the HemB-Class framework to predict the effects of mutations at FIXa positions not used during the training phase; thus, we created an index—the Severity Score—to quantify the likelihood that mutations cause a major disruption of the FIXa activity. In particular, we used the Severity Score to disambiguate several clinical HB reports that had conflicting observations, indicating that the HemB-Class captured in silico the essence of the FIXa structure (Figure 4A; Supplementary Table S5).

Finally, we explored the fullest extent of HemB-Class’ predictive power to study the effects of mutations of all FIXa residues to all 19 remaining amino acids (Figure 4B). This analysis produced a comprehensive list of residues that are likely intolerant to substitutions due to their high number of molecular interactions to other residues, their buried position and their evolutionary characteristics (Figure 4C; Supplementary Table S6); on the other hand, we found positions located at the outer loops of all FIXa domains that are more likely to accept amino acid substitutions, and are unlikely to disrupt the delicate inter-molecular network that holds the FIXa structure in place (del Sol et al., 2006; Han, 2008).

In summary, the FIXa RIN and the HemB-Class are versatile resources that can capture the intrinsic properties of the FIXa structure, and associate its features to the severity of HB. Thanks to its open source and scalable architecture, they can be immediately refined as soon as new FIX mutation reports and structures become available. Thus, we are optimistic that the findings presented here will pave the way for the rational design of better therapeutics, and that the overall methodology will be a starting point to study the underlying molecular mechanisms of other rare diseases.

## 4 Methods

### 4.1 Database Sanitation

We manually queried the European Association for Haemophilia and Allied Disorders Database (EAHAD) on 20th February 2021. At present, the EAHAD is the largest source of information about hemophilia B mutation in the public domain. It is manually curated and contains both clinical and genetic information (Rallapalli et al., 2013). We selected “Point” and “Polymorphism” (on type), and “Missense” (on variant effect) on the advanced search. Next, we removed mutations on the signal peptide regions, or outside the activated form of the protein.

Finally, we removed instances with ambiguous reported classifications (e.g., “mild/moderate,” or “moderate/severe”).

### 4.2 Creation of the FIXa Residue Interaction Network

To create a homology model of FIXa, the authors from a previous study (Rallapalli et al., 2013) aligned the structures of its individual domains (Freedman et al., 1995; Rao et al., 1995; Johnson et al., 2010) and the complete porcine (Brandstetter et al., 1995) version of FIX, and further refined its structure using optimization software. The domains and their resolutions were Gla: 2.80 Å, EGF1: 1.50 Å, EGF2-SP: 1.7 Å (PDB codes: 1CF1, 1EDM, and 3KCG, respectively).

We transformed the structure of the FIXa protein in an undirected, unweighted graph using RINerator version 0.5.1 (Doncheva et al., 2011) with the default parameters. This program first adds hydrogen atoms to the structure, which is essential to identify non-covalent interactions between amino acids (Word et al., 1999b), and second, it identifies the non-covalent interactions using a small probe (∼0.25 Å), rolled around the van der Waals surface of each amino acid (Word et al., 1999a), and a contact is established if the probe is simultaneously in contact with two non-covalently bonded atoms.

We considered that two residues interacted if there was at least one edge between them, independently of the edge type. To analyze the FIXa-RIN, we used R version 3.6.3 (https://www.R-project.org/) and the iGraph package, version 1.2.5 (Csardi and Nepusz, 2006). With the iGraph package, we used the function simplify to remove redundant edges and self-interactions. We calculated the degree, betweenness, closeness, Burt’s constraint (Burt, 2009), Authority Score, Page Rank-like, and the Authority Score measures.

We visualized the networks using Cytoscape version 3.8.2 (Shannon et al., 2003).

Finally, we obtained the conservation score from the ConsurfDB webserver (Ben Chorin et al., 2020), using the FIXa protein structure as input for the search query.

### 4.3 Calculation of the FIXa Protein Structure Properties

We used Chimera version 1.14 (Pettersen et al., 2004) to extract the solvent-excluded area (areaSES) and to calculate the relative surface exposure of all amino acids from the customized FIXa structure. We divided the solvent-excluded area of the residue by the surface area of the same type of residue in a reference state; in our case, we used the reference values of the 20 standard amino acids in Gly-X-Gly tripeptides (Bendell et al., 2014). Other measures calculated with Chimera were areaSAS, kdHydrophobicity, PSI, and PHI, for each residue of the FIXa structure.

To predict the secondary structure elements, we used the FIXa sequence as input to STRIDE (Frishman and Argos, 1995), and to determine whether the residue was buried of exposed, we divided the relative surface exposure area (relSESA) of each amino acid by the maximum value of all FIXa residues. Values below 0.25 were considered buried, otherwise, they were considered exposed.

For other measures, we used 393 FIXa single-point mutations as input to SIFT (Sim et al., 2012), Provean (Choi and Chan, 2015), and from Polyphen-2 (Adzhubei et al., 2010). For SIFT, we used the swiss_prot_2010_09 database, and a value of 0 for the median conservation of sequences.

### 4.4 Amino Acid Distance Index

We used the R package seqinR (Charif and Lobry, 2007) to obtain 544 numerical properties of each amino acid. Next, we used the package AMAP (Lucas, 2014) to perform a principal component analysis (PCA) of this set, and reduced the number of properties to 19 components while retaining 99% of the information in the dataset. Next, we calculated the Euclidean distance between all amino acid, considering all 19 component values. This gave us a 20 x 20 matrix which was the distance index used in our analyses (Supplementary Table S1).

#### 4.4.1 Machine Learning

We used supervised machine learning (ML) algorithms to analyze instances of FIXa mutations (input space χ) to predict different HB severities (output labels Ƴ). The learning process was executed in three steps. First (preprocessing), we organized the input space χ to be used as input for the ML algorithms. We removed all instances with a missing value in any FIXa feature. Then, we normalized the FIXa features to the interval [0, 1]. Next, we used a stratified 10-fold cross-validation strategy to find the best possible ML models from the mapping f: χ→Ƴ. This strategy randomly splits the input space in ten parts, respecting the original distribution of the output labels (213 mild/moderate and 180 severe). During this phase, the optimal ML models (considering different ML algorithms) are obtained by iteratively using 9 folds for training and 1 fold to evaluate the output of the algorithms. Finally, the final performance of such models was assessed using validation methods to compare the expected and the predicted HB severity for a set of unseen examples. The last two steps were repeated 10 times to ensure that the results were consistent despite random fluctuations. The validation methods used here were the accuracy, Kappa Coefficient, Matthews Correlation Coefficient (MCC), and Area under the ROC curve (AUC). The accuracy is used to determine the number of instances classified correctly. The Kappa Coefficient measures the agreement between the predicted and the expected severity, emphasizing that the results were not obtained by chance. The MCC uses a contingency matrix, produced by the expected and the predicted severity, to compare classifiers in a way similar to the Pearson’s correlation coefficient. Finally, the AUC uses a contingence matrix to create a curve between the TPR (True Positive Rate) and FPR (False Positive Rate) values. As the area under the curve approaches 1, the quality of the classification increases.

The ML algorithms used in our study were: Decision Trees (DT) (Breiman, 1984), XGBoost (Chen and Guestrin, 2016), Random Forest (RF) (Breiman, 2001), and Support Vector Machine (SVM) (Vapnik, 2000). Our ensemble was built on top of Random Forest and XGBoost, whose combination provided the best results.

For all ML algorithms, the training step relied on a grid search strategy to determine the best parametrization. The DT model was optimized by varying the minimum number of observations in a node before splitting the data within the interval [2, 50]. The minimum number of observations in a terminal node (leaf) was searched in the interval [1, 35]. Finally, we looked for the optimal complexity parameter (cp) within the range [0.0001, 1]. We trained the Random Forest (RF) model by varying the number of trees (ntree) in the interval [4,100], the number of variables randomly sampled as candidates at each split (mtry) in the interval [2, 7], and minimum size of terminal nodes or leaves (nodesize) between [1, 5]. The Naïve Bayes model was estimated by only varying the Laplace smoothing, to avoid handling with zero probabilities, in the interval [0, 1]. The SVM (Support Vector Machine) models were adjusted using the two best kernels: radial $\epsilon^{\left(-y{\left[x-\omega \right]}^{2}\right)}$ , and polynomial ${\left(y\omega \prime x+c\right)}^{d}$ , such that *x* is a vector representing the training data and ω is the kernel coefficient varying in [0, 0.1, 0.2, ..., 2]. For the radial kernel, we analyzed the following parameters *y* = [0.01, 0.02, ..., 1.5], while the polynomial kernel was assessed using *c* = [0, 0.1, 0.2, ..., 2], *d* = {2, 3, 4, 5}. Finally, XGBoost was optimized considering the maximum depth of a tree in the interval [1, … , 25], and the learning rate (parameter ɳ) in the interval [0, 0.5].

In our experiments, we used the R statistical package 3.6.3 and the MLR package (Bischl et al., 2016) (version 2.19.0), which provides a machine learning interface to train models by using hyperparameter tuning, cross validation, feature selection, ensemble construction, and model validation. Internally, the MLR package calls the e1071 package (version 1.4.1.1 - https://cran.r-project.org/web/packages/e1071/index.html) to create the SVM model, the XGBoost package (v1.7-6) (Chen and Guestrin, 2016) to create the ensemble model using the gradient boosting approach, and the rpart package (v4.1-15) (Breiman, 1984) to create the DT model. All packages are available at the CRAN repository (https://cran.r-project.org).

## Data Availability

The original contributions presented in the study are included in the article/Supplementary Material, further inquiries can be directed to the corresponding author. The HemB-Class source code and datasets developed in this study are available at https://github.com/ricardoarios/HemB-Class.
