# Supplementary material for: A Machine Learning Framework Predicts the Clinical Severity of Hemophilia B Caused by Point-Mutations
Source: Front Bioinform. 2022 Jun 23;2:912112. doi: 10.3389/fbinf.2022.912112 (PMC9580853; doi:10.3389/fbinf.2022.912112)
Supplement: Supplementary file 1 [file DataSheet1.zip › Supplementary Figure 4 - Confusion matrices.pdf]

Provean	Deleterious	Neutral
Severe	166	14
Mild/Moderate	160	53

Provean	Damaging	Tolerated
Severe	170	10
Mild/Moderate	165	48

Polyphen	Benign	Possibly damaging	Probably damaging
Severe	9	14	157
Mild/Moderate	41	32	140

Polyphen	Deleterious	Neutral
Severe	170	10
Mild/Moderate	170	43
