# Supplementary material for: A Machine Learning Framework Predicts the Clinical Severity of Hemophilia B Caused by Point-Mutations
Source: Front Bioinform. 2022 Jun 23;2:912112. doi: 10.3389/fbinf.2022.912112 (PMC9580853; doi:10.3389/fbinf.2022.912112)
Supplement: Supplementary file 1 [file DataSheet1.zip › Supplementary Material - Final.docx]

**Supplementary Material**

**Supplementary Figure 1: Comparison to the AlphaFold2 Model.**

(A) The model confidence displayed for the FIX protein structure model predicted by AlphaFold 2 and the researchers from the European Bioinformatics Institute (EBI) (Tunyasuvunakool et al., 2021). (B) The structural alignment of the model we used here, derived from a previous study (Rallapalli et al., 2013), and the AlphaFold 2 structure. Overall, the distance is ~1.85 Å. The structural alignment was performed using Chimera version 1.14 (Pettersen et al., 2004).

**Supplementary Figure 2: Ramachadran plot**

Depicted is the Ramachadran plot of the FIXa structure. Here, highly preferred observations (green) are 274 amino acids (87.26%), the preferred observations (orange) are 31 amino acids (9.87%), and questionable observations (red) are 9 residues (2.86%).

**Supplementary Figure 3: Analysis of hydrogen bonds and residue distances.**

The boxplot displays the distances in Å between two amino acids interacting via a hydrogen bond. All interactions are listed in the Supplementary Table 2. The boxplots show the median (center line), the first and third quartiles (lower- and upper-bounds), and 1.5 times the inter-quartile range (lower- and upper whiskers).

**Supplementary Figure 4 – Predictive power of existing methods.**

These confusion matrices show the relation between the classification outputted by Provean (Choi and Chan, 2015) and Polyphen-2 (Adzhubei et al., 2010), when considering 393 FIXa single-point mutations. Although in all cases there was a significant association (Fisher’s test, p-value < 0.01), the results are clearly incorrect. However, as shown in Figure 1c, we verified that the raw numerical scores outputted by these methods are powerful discriminators of the two hemophilia B severities (Mild/Moderate vs Severe).

**Supplementary Table 1: The structural and centrality measures of all residues of the FIXa protein.**

**Supplementary Table 2: The FIXa Residue Interaction Network**

**Supplementary Table 3: Residues involved in interdomain interactions.**

**Supplementary Table 4: Classification measures of the machine learning classifiers.**

**Supplementary Table 5: Predictions of the severity of mutations with conflicting results.**

**Supplementary Table 6: Predictions of the severity of all possible FIXa mutations.**
