# Supplementary figures and images for: A Machine Learning Framework Predicts the Clinical Severity of Hemophilia B Caused by Point-Mutations
Source: Front Bioinform. 2022 Jun 23;2:912112. doi: 10.3389/fbinf.2022.912112 (PMC9580853; doi:10.3389/fbinf.2022.912112)

**A**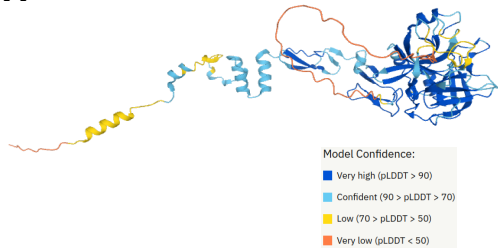**B**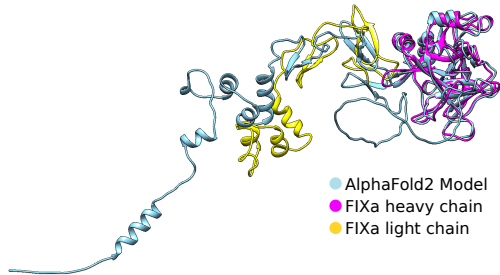

Supplement: Supplementary file 1 [file DataSheet1.zip › Supplementary Figure 1 - AF and FIX align.pdf]

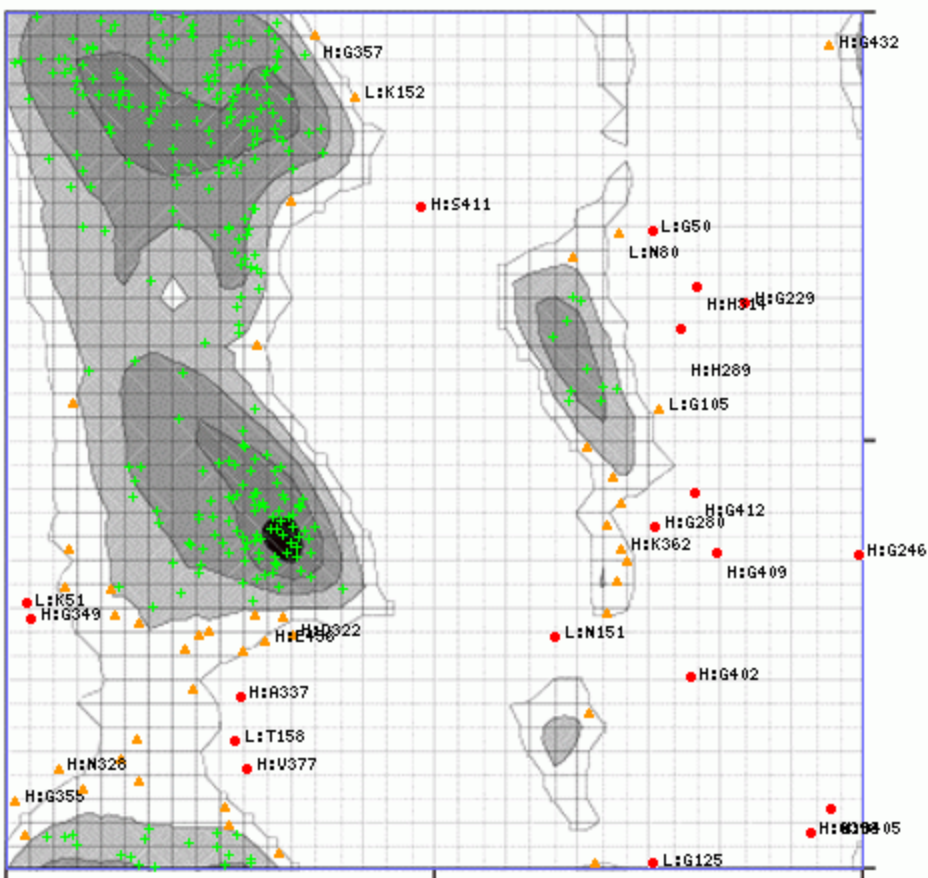

Supplement: Supplementary file 1 [file DataSheet1.zip › Supplementary Figure 2 - Ramachandran_plot.pdf]

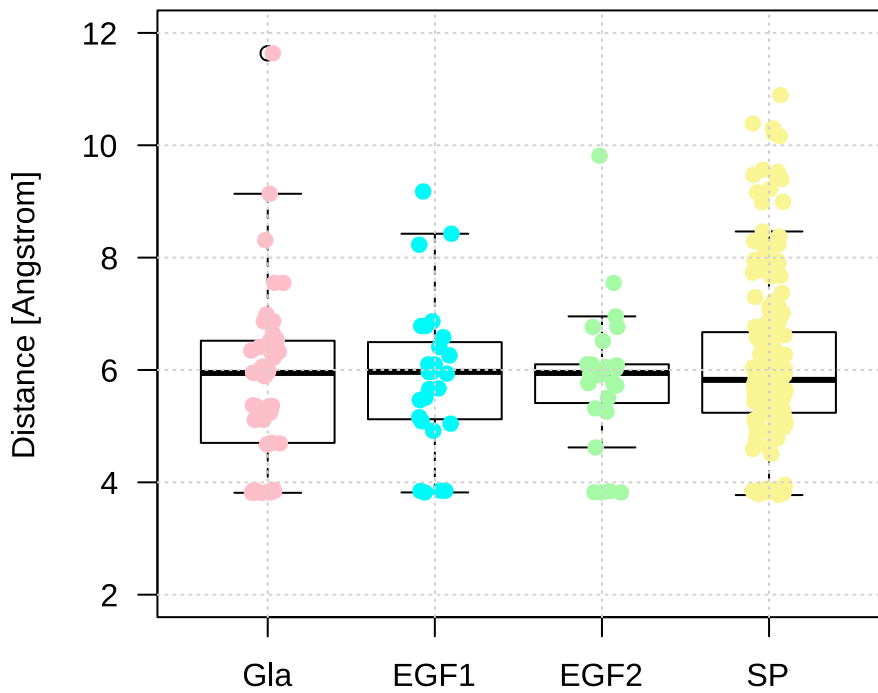

Supplement: Supplementary file 1 [file DataSheet1.zip › Supplementary Figure 3 - hbond_distances.pdf]
